# The different effects of twin boundary and grain boundary on reducing tension-compression yield asymmetry of Mg alloys

**DOI:** 10.1038/srep29283

**Published:** 2016-07-04

**Authors:** Huihui Yu, Yunchang Xin, Adrien Chapuis, Xiaoxu Huang, Renlong Xin, Qing Liu

**Affiliations:** 1 School of Materials Science and Engineering, Chongqing University, Chongqing 400044, People’s Republic of China

## Abstract

In the present study, a coarse grained AZ31 plate was refined by 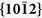 twin boundaries (TBs) and grain boundaries (GBs), respectively. A comparative study about the different effects of grain refinements by GBs and by TBs on tension-compression yield asymmetry was performed. Our results show that both the refinements by GBs and by TBs increase the tensile and compressive yield strengths, but to a different degree. 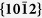 TBs are more effective to harden 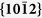 twinning, but yield a lower strengthening against prismatic <a> slip, and a much lower tension-compression yield asymmetry is thus obtained. Both the differences in boundary coherence and misorientation between GBs and TBs affect the hardening. The misorientation of TBs provides a lower geometric compatibility factor (a higher hardening) for both prismatic <a> slip and 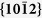 twinning than that of GBs, which in detail is the result of the much higher angle between c-axes of the two sides of TBs (about 86°) than GBs (0–50°). It is found that, for hardening of prismatic <a> slip, boundary coherence plays a more important role than misorientation. With regard to 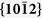 twinning, the different misorientation of TBs from GBs mainly accounts for their different hardening effects.

Mg alloys have aroused much attention as lightweight structural metals. A strong texture often develops after thermomechanical processing. One key problem with the application of highly textured Mg products is high levels of tension-compression yield asymmetry (Tensile yield strength (TYS) greatly differs from the compressive one (CYS) along the same direction)^1^. For example, CYS of Mg AZ31 plates along the transverse direction is often half of TYS^1^. The origin of this yield asymmetry is now well understood: the ease and polarity of 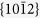 twinning^2^. 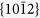 twinning with a low critical resolved shear stress (CRSS) is one of the mostly active deformation modes at room temperature^2^. The polarity of twinning dictates that, if 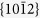 twinning dominates the compressive deformation, it has to give way to slip with a higher CRSS (e.g. prismatic <a> slip) during tension. Then, a tension-compression yield asymmetry would be generated.

Precipitation is found to be an effective way to reduce this yield asymmetry^3^,^4^,^5^. For example, Stanford *et al*. reported that the basal plate precipitates in AZ91 can increase the value of CYS/TYS from 0.75 for the solid soluted sample to 0.91 for the aged one^5^. The reason is attributed to the preferentially hardening against 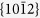 twinning by precipitates in comparison with prismatic <a> slip^6^. However, it is inappropriate to hold that precipitates are always helpful to reduce the yield asymmetry without considering their shapes and habits. Robson *et al*. found that the rod precipitates parallel to the c-axis in Mg-Zn system hardly reduce the yield asymmetry^6^.

Grain refinement is also effective to decrease this yield asymmetry^7^. A CYS/TYS of 0.4–0.5 often exists in coarse-grained Mg alloys^1^, while increases to 0.9 with refining grain size to 1.9 μm. Although grain refinement would enhance the CRSSs for both slip and twinning, that for twinning often increases to a larger extent^8^. Besides grain boundaries (GBs), twin boundaries (TBs) can also be employed to refine grains^9^. The authors^10^,^11^ reported that numerous 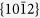 TBs can harden both slip and 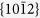 twinning. However, it is considered that a coherent TB may pose different effects from a GB against dislocation motion^11^. In addition, the misorientation angle of 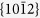 TBs is about 86°, much higher than that of GBs (a large fraction between 10°–40°) in basal textured plates. Therefore, it is speculated that grain refinements by GBs and by TBs would pose different effects on reducing yield asymmetry. However, there is no comparative study addressing this effect. In this work, we reported a higher efficiency of TBs than GBs in reducing tension-compression yield asymmetry of an AZ31 plate. Our results further uncovered that the two types of boundaries generated quite different hardening against not only prismatic <a> slip, but 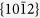 twinning. The relevant mechanisms were studied and discussed.

## Results

### Microstructure examined by EBSD

**Figure 1:**
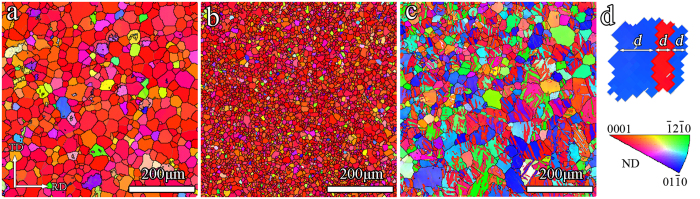
Inverse pole figure maps of (**a**) GB-coarse, (**b**) GB-refine and (**c**) TB-refine; (**d**) showing the method to measure the average of lamellae spacing (*d*) in TB-refine.

Figure 1 shows the inverse pole figure maps of three types of sample. Both the GB-coarse and GB-refine have a fully recrystallized structure, with an average grain size of about 35 μm for GB-coarse and 8.1 μm for GB-refine. A large number of twin bands identified as 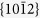 twins exist in TB-refine (Fig. 1c). When grains are subdivided by twin lamellae, the lamella spacing can be considered as the equivalent grain size^12^,^13^. Using the method shown in Fig. 1d, the average grain size in TB-refine was measured to be 8.7 μm, similar to that in GB-refine (8.1 μm).

### Tension-compression yield asymmetry

**Figure 2:**
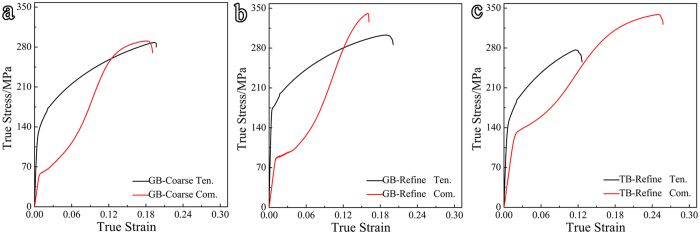
True stress-strain curves under tension and compression along the TD of (**a**) GB-coarse, (**b**) GB-refine and (**c**) TB-refine. Ten. and Com. denote tensile and compressive curves, respectively.

True stress-strain curves under tension and compression along the TD are plotted in Fig. 2. All compressive curves have a plateau, the typical feature of a 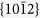 twinning predominant deformation^14^. Yield strengths derived from those curves are listed in Table 1. Both the refinements by GBs and by TBs increase the tensile and compressive yield strengths, but to a different degree. For example, GBs refinement increases TYS by 50 MPa, while TBs refinement 20 MPa. In contrast, TBs refinement improves CYS to a higher degree. The tension-compression yield asymmetry measured as CYS/TYS in GB-refine (0.5) is much higher than that in TB-refine (0.86). That is, grain refinement by TBs is much more effective to reduce this yield asymmetry.

### Texture and Schmid factor

**Figure 3:**
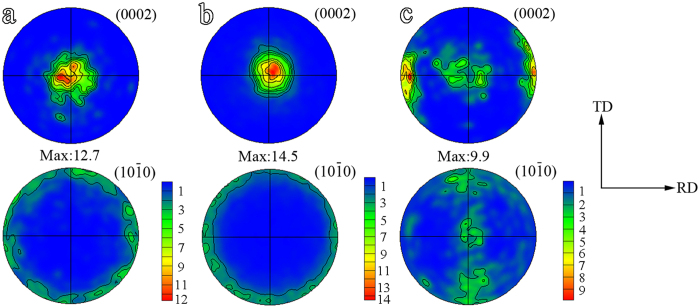
Pole figures of (**a**) GB-coarse, (**b**) GB-refine and (**c**) TB-refine.

Pole figures of the three samples are given in Fig. 3. Both the GB-coarse and GB-refine have a typical basal texture with (0002) poles largely parallel to the ND. There is no preferred orientation for the prismatic planes. TB-refine contains two texture components, basal poles largely parallel to the ND and RD, respectively. As 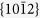 twinning generally rotates the basal poles by about 86° toward the compression direction^15^, the (0002) poles close to the RD come from 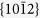 twins and those around ND the matrix.

**Figure 4:**
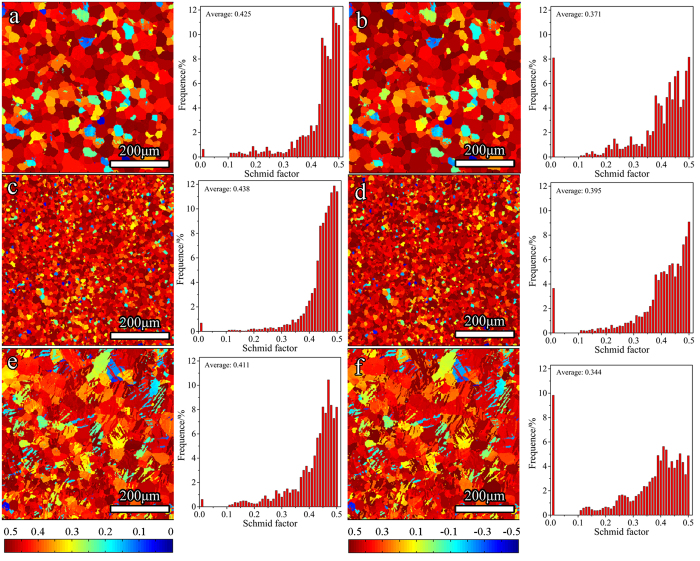
Schmid factors (SFs) as a function of relative spatial position and relative distributions for prismatic <a> slip under tension along the TD and 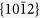 twinning under compression along the TD: (**a**) prismatic <a> slip and (**b**) 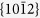 twinning in GB-coarse; (**c**) prismatic <a> slip and (**d**) 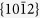 twinning in GB-refine; (**e**) prismatic <a> slip and (**f**) 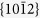 twinning in TB-refine. Note that 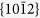 twinning whose SF is negative would lead to contraction along the c-axis and not be activated. A negative value of SF for 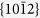 twinning is therefore treated as zero during calculation of the distribution and the average of SFs.

Although TB-refine has a texture different from the GB-coarse or GB-refine, according to previous studies, the orientations in the three samples favor prismatic <a> slip under tension along the TD, and 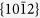 twinning during compression^1^,^11^. To further confirm this, Schmid factors (SFs) in the three samples under tension and compression along the TD were calculated, with the results shown in Fig. 4. Obviously, there are high and similar SFs for prismatic <a> slip in the three samples, so do the SFs for 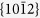 twinning. Therefore, the same deformation mechaism will be initiated in the three samples during tension or compression along the TD.

## Discussion

The efficiency of grain refinement on reducing tension-compression yield asymmetry is mainly determined by the difference between the hardening against 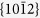 twinning and that against prismatic <a> slip. When twinning is more effectively hardened than slip, it would generate a pronounced reduction in yield asymmetry. As evidenced from Table 1, grain refinement by TBs is more effective to hardening 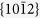 twinning than GB refinement, while GBs refinement has a higher efficiency in hardening prismatic <a> slip. Therefore, TBs show a much higher efficiency in reducing yield asymmetry. Now, there comes a question why there is a difference in hardening against 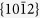 twinning and prismatic <a> slip between GBs and TBs. The effect of grain refinement on yield strength (*σ*_*y*_) can be well predicted by the Hall-Petch relationship^16^:


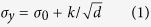


where *σ*_0_ is the friction stress when dislocations move on the slip plane, *d* is the average grain size and *k* the stress concentration factor. According to the Hall-Petch relationship, yield strength is determined by yielding of the grain interior (*σ*_0_ = *Mτ*_0_) and boundary obstacle effect (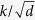), where *M* is the Taylor orientation factor and *τ*_0_ the CRSS. *M* can be calculated as the reciprocal of averaged SF^17^. As seen in Fig. 4, the mean SFs for either the prismatic <a> slip or 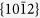 twinning in TB-refine are similar to those in GB-refine and GB-coarse and thus, *σ*_0_ in the three samples is similar. That is, the different hardening effects of TBs from GBs in this study mainly result from their different boundary obstacle effects.

**Figure 5:**
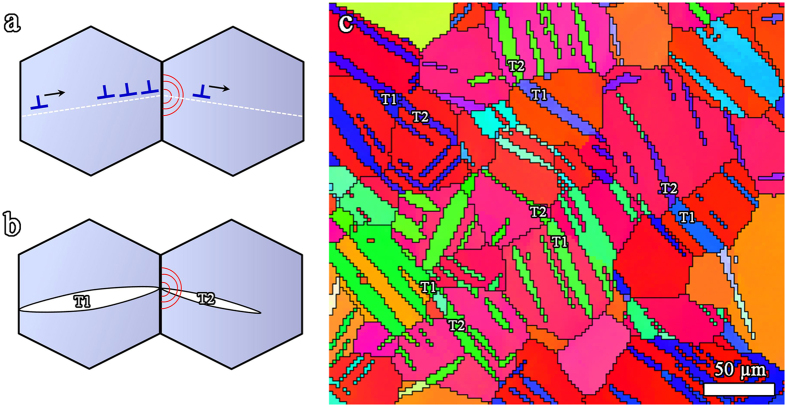
A schematic diagram showing (**a**) slip or (**b**) twinning propagation from one grain to the neighboring one; (**c**) an inverse pole figure map showing 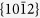 twin (T) transfer between two neighbored grains in a twinned AZ31 plate, forming paired twins T1-T2.

As schematically illustrated in Fig. 5a, the boundary obstacle effect on slip is well understood in terms of dislocations pile-up in the vicinity of GBs. The yielding happens when the pile-up of dislocations exerts sufficient stress at GBs to generate slip propagation from one grain to its neighbor^18^. Up to now, the boundary obstacle effect on twinning is not well understood. To maintain homogeneous deformation, twinning transfer between neighbored grains is necessary. Barnett *et al*. reported that twinning did not occur in all grains simultaneously. The twin nucleation, propagation and transfer to neighbor grains dominate the initial yielding during a 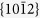 twinning predominant deformation of Mg AZ31^19^. Direct observation and simulation suggest that twinning generally nucleates at GB followed by a fast propagation and termination at the next GB^19^. The termination of a twin at a GB will generate a localized stress concentration which would stimulate and trigger twin nucleation in next grain (see Fig. 5b). This type of twinning transfer between neighbored grains is extensively observed in twinned Mg AZ31^20^, forming paired twins (T1-T2) similar to that in Fig. 5c. Therefore, the boundary obstacle effect on twinning can be described by the effect of boundary on twinning transfer between neighbored grains.

The boundary obstacle effect is mainly associated with the boundary misorientation and boundary structure (e.g. coherent or incoherent). Compared to a non-coherent boundary, a coherent one seems to serve as a weaker barrier for dislocation penetration^11^,^21^,^22^,^23^. Therefore, the coherent structure of TBs would generate a lower obstacle effect on prismatic <a> slip than the incoherent structure of GBs. As twinning transfer between grains mainly involves the effect of stress concentration by twin termination at boundary on twin nucleation in the next grain, boundary coherence is considered to hardly affect twinning transfer. A GB might possess a boundary misorientation different from a TB, which would pose an effect on boundary obstacle effect. The usage of geometric compatibility factor (*m*′) to evaluate the effect of boundary misorientation on slip or twinning transfer between neighbored grains has been extensively reported^17^,^19^,^24^,^25^,^26^,^27^:


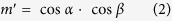


where *α*/*β* is the angle between the slip (twinning) planes/slip (twinning) directions of two neighbored grains. The *m*′ varies between 0 and 1. For *m*′ = 1, both the slip planes and the slip directions are parallel. In this case, deformation would be expected to easily propagate from one grain to the next one, as slip transfer has no need to change both the slip plane and the slip direction. In contrast, *m*′ = 0 indicates that either slip directions or slip planes are orthogonal, leading to a completely incompatible condition for slip transfer at boundary. A higher *m*′ often indicates a lower boundary obstacle effect and *vice versa*. Previously, the *m*′ is generally used to investigate deformation behavior in two neighbored grains. As the value of *m*′ represents the difficulty of boundary on deformation transfer between neighbored grains, it can be used to evaluate this boundary obstacle effect on yield strength.

**Figure 6:**
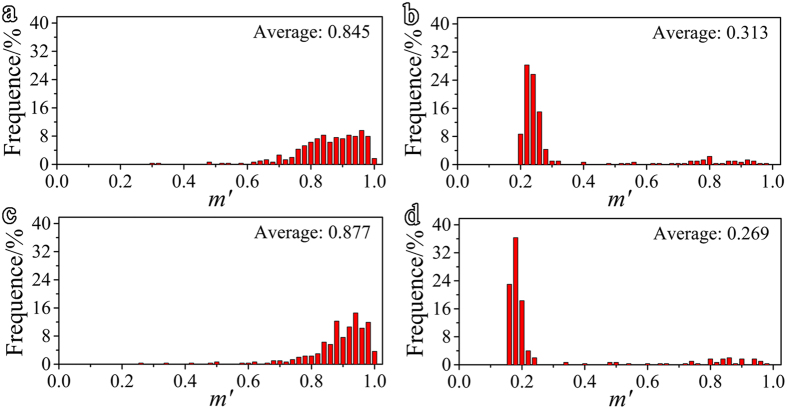
The distribution of the geometrical compatibility factor (*m*′) for prismatic <a> slip transfer in (**a**) GB-refined sample, (**b**) TB-refined sample and for 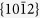 twinning transfer in (**c**) GB-refined sample and (**d**) TB-refined sample.

The distribution of *m*′ for prismatic <a> slip and 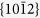 twinning calculated from randomly selected 400 pairs of neighbored grains in EBSD data is given in Fig. 6. For prismatic <a> slip, the *m*′ was calculated between each prismatic <a> slip system in one grain (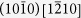, 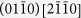 and 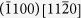) and that of its adjacent grain. As dislocations can slid in two opposite directions, a negative value of *m*′ indicates the effect on dislocation sliding in the opposite direction. Therefore, the maximum absolute value of *m*′ is used to represent the geometric compatibility for prismatic <a> slip. For 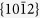 twinning, the *m*′ between each 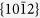 twinning variant in one grain (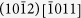, 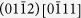, 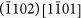, 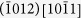, 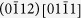 and 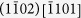) and that in its adjacent grain is calculated. As twinning only allows shear in one direction, a negative value of *m*′ indicates a completely incompatible twinning transfer. The maximum *m*′ is used to indicate the geometric compatibility for twinning transfer between two grains. As seen in Fig. 6a,b, the majority of *m*′ for prismatic <a> slip in GB-refine (average 0.845) are higher than 0.8, whereas those in TB-refine (average 0.313) mainly fall within 0.2–0.32. Similarly, the average of *m*′ for 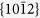 twinning in GB-refine (0.877) is much higher than that in TB-refine (0.269).

As discussed above, both the differences in boundary coherence and misorientation between GBs and TBs affect their hardening on slip and twinning. The difference in boundary misorientation is in essence a texture difference. As evidenced from Fig. 6, the misorientation of TBs provides a lower *m*′ for prismatic <a> slip than that of GBs and, hence, a higher hardening effect. However, the coherent structure of TBs yields a lower hardening. The experimental results in Table 1 clearly indicate that TBs exert a lower hardening against prismatic <a> slip. This is an indication that boundary coherence poses a more influential effect on hardening against prismatic <a> slip. With regard to 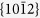 twinning, boundary coherence hardly affects its hardening effect. The misorientation of TBs leads to a lower *m*′ and, thus, a higher hardening, which agrees well with the results in Table 1. Therefore, the different misorientation of TBs from GBs mainly accounts for their different hardening effects.

**Figure 7:**
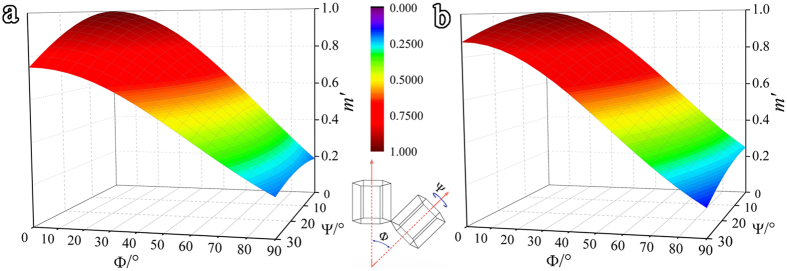
The maximum geometrical compatibility factor (*m*′) for (**a**) prismatic <a> slip transfer and (**b**) 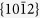 twinning transfer as a function of the tilting angle of c-axes between two neighbored grains (Φ) and the rotation angle around the c-axis (ψ).

**Figure 8:**
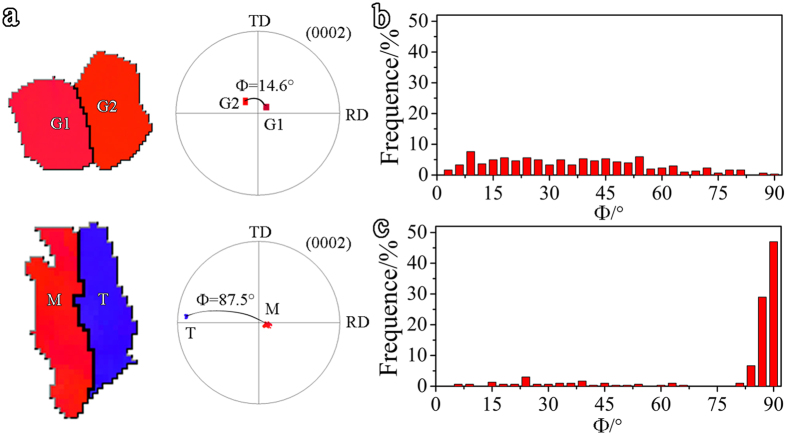
(**a**) Diagrams showing the method to measure Φ between two neighboring grains (G1 and G2) and that between the matrix (M) and its neighboring twin (T); the distribution of Φ in (**b**) GB-refine and (**c**) TB-refine.

It is interesting to know that why there is a lower *m*′ for 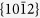 twinning or prismatic <a> slip in TB-refine than GB-refine. To answer this question, it is important to know the relationship between *m*′ and boundary misorientations. This analysis is presented in Fig. 7. The insert in Fig. 7a shows that the orientation relationship of two neighbored grains can be described as an angle between their c-axes (Φ) and a rotation around c-axis (ψ). Due to the symmetry of the *hcp* structure, ψ varies between 0°–30° and Φ between 0°–90°. For both prismatic <a> slip and 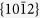 twinning, *m*′ drops quickly with increasing Φ, whereas varying ψ at a given Φ only slightly changes *m*′. The value of *m*′ is therefore mainly determined by the angle between the c-axes of two grains. For both TB-refine and GB-refine, the Φ of 400 pairs of neighbored grains was measured and shown in Fig. 8. As seen in Fig. 8a, Φ in GB-refine is the measured angle between the (0002) poles of two neighbored grains, while, in TB-refine, besides Φ between neighbored grains, Φ between two sides of a TB is also included. The Φ in GB-refine has a broad distribution between 0–50°, while the majority of Φ in TB-refine are higher than 80°. As 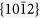 twinning rotates the basal poles by about 86°, the Φ higher than 80° in TB-refine mainly comes from twin-matrix boundaries, TBs. Therefore, a large number of 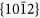 TBs are the main reason for the much lower *m*′ for 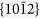 twinning in TB-refine.

## Conclusion

In the present study, a coarse grained AZ31 plate was refined by two different types of boundary (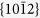 TBs and GBs), respectively. A comparative study about the different effects of grain refinements by GBs and by TBs on tension-compression yield asymmetry was carried out. The mechanisms for the different hardening effects between GBs and TBs were systematically studied. Several conclusions are reached as follows:

(1) Both the grain refinements by GBs and by TBs increase the tensile and compressive yield strengths, but to a different degree. TBs are more effective to harden 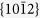 twinning, but yield a lower strengthening against prismatic <a> slip. A much lower tension-compression yield asymmetry in the TB-refined sample than the GB-refined one is thus obtained.

(2) Both the differences in boundary coherence and misorientation between GBs and TBs affect the hardening effect. The misorientation of TBs provides a lower *m*′ for both prismatic <a> slip and 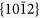 twinning than that of GBs. For hardening of prismatic <a> slip, the boundary coherence plays a more influential role than misorientation. With regard to 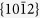 twinning, the misorientation of TBs than GBs mainly accounts for their different hardening effects.

(3) The lower *m*′ for both prismatic <a> slip and 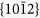 twinning in TB-refined sample than that in the GB-refined one is mainly originated from the much higher angle between c-axes of the two sides of TBs (about 86°) than GBs (0–50°).

## Methods

### Sample preparation

A Mg AZ31 plate with a grain size 35 μm (the designated GB-coarse) was used. The GB-coarse was hot rolled at 400 °C to 25% reduction and subsequently annealed at 250 °C for 4 h to prepare the sample refined by GBs (the designated GB-refine with an average grain size 8.1 μm). To fabricate the sample refined by 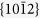 TBs (the designated TB-refine), the GB-coarse plate (10 mm (RD) × 80 mm (TD) × 10 mm (ND)) was pre-rolled at room temperature, with 3% thickness reduction along the RD followed by annealing at 200 °C for 2 h to reduce the dislocations density. Here, RD, TD and ND refer to the rolling direction, transverse direction and normal direction of the initial plate, respectively.

### Mechanical tests

Tension and compression tests along the TD at room temperature were carried out on a Shimadzu AG-X machine at a strain rate of 0.001 s^−1^. The specimens for compression tests were blocks of 9 mm (RD) × 9 mm (ND) × 12 mm (TD), and those for tension test were dog bone shape with 13 mm in gauge length and 4 × 2.5 mm in cross section. Each mechanical test was repeated three times to get representative results.

### Microstructure and texture examination

Microstructure and crystallographic orientations were analyzed by an electron back-scattered diffraction (EBSD) technique. EBSD mapping was conducted on a scanning electron microscope (SEM, TESCAN MIRA3) equipped with a HKL-EBSD system using a step size of 1.5 μm. The samples for EBSD mapping were mechanical ground followed by electro-chemical polishing in an AC2 electrolyte solution at 20 V for 90 s. Data were acquired and post-processed using HKL Channel 5 software.

## Additional Information

**How to cite this article**: Yu, H. *et al*. The different effects of twin boundary and grain boundary on reducing tension-compression yield asymmetry of Mg alloys. *Sci. Rep.* **6**, 29283; doi: 10.1038/srep29283 (2016).

## Figures and Tables

**Table 1:** Yield strength of different samples under tension and compression along the TD.

Sample	CYS/MPa	TYS/MPa	CYS/TYS
GB-coarse	56 ± 2	127 ± 3	0.44
GB-refined	89 ± 2	177 ± 6	0.50
TB-refined	126 ± 3	147 ± 8	0.86

CYS and TYS represent the yield strength under compression and that under tension, respectively.
